# Feasibility and acceptability of a mindfulness app-based intervention among patients with metastatic renal cell carcinoma: a multinational study

**DOI:** 10.1093/oncolo/oyae309

**Published:** 2025-01-17

**Authors:** Cristiane Decat Bergerot, Paulo Gustavo Bergerot, Errol J Philip, Jasnoor Malhotra, Daniela V Castro, Ameish Govindarajan, William Hiromi Fuzita, Marcos Vinicius da Silva França, Andressa Cardoso de Azeredo, Gabriel Marques dos Anjos, Romildo de Araujo, JoAnn Hsu, Neal Chawla, Alex Chehrazi-Raffle, Marco Murilo Buso, Bechara Saab, Linda E Carlson, Sumanta K Pal

**Affiliations:** Oncoclinicas&Co—Medica Scientia Innovation Research (MEDSIR), Sao Paulo, SP 04543-906, Brazil; Oncoclinicas&Co—Medica Scientia Innovation Research (MEDSIR), Sao Paulo, SP 04543-906, Brazil; University of California Los Angeles, Los Angeles, CA 90095, United States; Department of Medical Oncology & Experimental Therapeutics, City of Hope Comprehensive Cancer Center, Duarte, CA 91010, United States; Department of Medical Oncology & Experimental Therapeutics, City of Hope Comprehensive Cancer Center, Duarte, CA 91010, United States; Memorial Sloan Kettering Cancer Center, New York, NY 10065, United States; Oncoclinicas&Co—Medica Scientia Innovation Research (MEDSIR), Sao Paulo, SP 04543-906, Brazil; Oncoclinicas&Co—Medica Scientia Innovation Research (MEDSIR), Sao Paulo, SP 04543-906, Brazil; Oncoclinicas&Co—Medica Scientia Innovation Research (MEDSIR), Sao Paulo, SP 04543-906, Brazil; Oncoclinicas&Co—Medica Scientia Innovation Research (MEDSIR), Sao Paulo, SP 04543-906, Brazil; Oncoclinicas&Co—Medica Scientia Innovation Research (MEDSIR), Sao Paulo, SP 04543-906, Brazil; Department of Medical Oncology & Experimental Therapeutics, City of Hope Comprehensive Cancer Center, Duarte, CA 91010, United States; Department of Medical Oncology & Experimental Therapeutics, City of Hope Comprehensive Cancer Center, Duarte, CA 91010, United States; Department of Medical Oncology & Experimental Therapeutics, City of Hope Comprehensive Cancer Center, Duarte, CA 91010, United States; Oncoclinicas&Co—Medica Scientia Innovation Research (MEDSIR), Sao Paulo, SP 04543-906, Brazil; Mobio Interactive Pte. Ltd., Gateway West 189720, Singapore; Department of Oncology, University of Calgary Cumming School of Medicine, Calgary, Alberta T2N 4N1, Canada; Department of Medical Oncology & Experimental Therapeutics, City of Hope Comprehensive Cancer Center, Duarte, CA 91010, United States

**Keywords:** kidney cancer, digital health, mindfulness, mind-body therapies, emotional distress, fatigue

## Abstract

**Background:**

Patients with metastatic renal cell carcinoma (mRCC) experience emotional distress and limited supportive care access. This study assesses a mindfulness app’s feasibility, acceptability, and preliminary efficacy in improving emotional symptoms, trait mindfulness, and overall quality of life for patients with mRCC on immunotherapy.

**Methods:**

This multinational study recruited patients with mRCC undergoing immunotherapy from Brazil and the United States. Participants were required to engage in mindfulness app-based activities for 20–30 min daily, at least 4 days per week, over a 4-week period. Assessments were conducted at weeks 0, 2, 4, and 12 to evaluate emotional symptoms (PROMIS-Anxiety and Depression, Fear of Cancer Recurrence-7), fatigue (Brief Fatigue Inventory), trait mindfulness (Mindfulness Attention Awareness Scale), and quality of life (Functional Assessment of Chronic Illness Therapy-General). Self-reported data were used to assess adherence. Linear mixed-effects models were used to evaluate changes over time for the measured outcomes.

**Results:**

Among 50 patients with mRCC, the feasibility of this intervention was demonstrated; 96% of patients were assessed at week 4, with high adherence rates reported by 75% of patients. Participants expressed positive feedback on the smartphone-based approach. Significant improvements were observed in emotional symptoms, fatigue, and quality of life scores from baseline to post-intervention (*P* = .001 for each), suggesting the positive impact of this intervention.

**Conclusion:**

Our findings provide encouraging evidence for the feasibility and acceptability of a mindfulness app-based intervention among patients with mRCC. This intervention may offer a viable and accessible means of providing psychosocial support to patients with mRCC.

**Key points**

- We evaluated a mindfulness app for feasibility, acceptability, and initial impact on emotional distress and quality of life in patients with mRCC on immunotherapy.
- Patients from Brazil and the U.S. used the app 20-30 min/day, 4 days/week over 4 weeks. Key measures included emotional symptoms, fatigue, and quality of life.

**Key Findings**

- High adherence: 96% completed assessments, 75% consistently engaged with the app.
- Significant improvements: Reductions in emotional distress and fatigue, improved quality of life (P = 0.001).
- Implication: Mindfulness apps can provide accessible, effective psychosocial support for patients with mRCC, enhancing care beyond traditional in-person support.

**Implications for practice**

In a multinational study with 50 metastatic renal cell carcinoma patients on immunotherapy, a mindfulness app-based intervention demonstrated 96% feasibility and 75% adherence, showing significant improvements in emotional symptoms, fatigue, and quality of life (*P* = .001 each). This accessible tool holds promise for alleviating distress and enhancing well-being, supporting potential integration into clinical care.

## Introduction

Despite expanding treatment options for patients diagnosed with metastatic renal cell carcinoma (mRCC), those diagnosed still face a disease trajectory marked by emotional and physical symptoms, treatment side effects, and impairment in health-related quality of life (HRQOL).^1^ As a result, considerable efforts have been devoted to developing effective and easily accessible psychological interventions that can alleviate emotional distress and promote effective coping with physical symptoms.

Mindfulness-based interventions (MBIs) interventions have shown promise for reducing emotional distress in the form of anxiety, depression, and fear of cancer recurrence or progression, as well as physical symptoms including fatigue, insomnia, and pain, thereby improving overall HRQOL.^2–9^ MBI programs typically include a range of practices that introduce formal exercises such as body scans (guided attentional exercise throughout the body), sitting meditation, and mindful awareness into daily routines and activities.^10^ The duration of MBI programs varies, lasting from 6 to 8 weeks, with approximately 2-hour weekly group sessions and daily home practice.^10^

One example is the Mindfulness-Based Cancer Recovery program, a 9-week group behavioral treatment program aimed at mitigating emotional and physical symptoms while enhancing overall HRQOL and social support.^11,12^ In a large clinical trial comparing 2 group interventions, MBCR and supportive-expressive group therapy, MBCR showed superior effectiveness in lowering stress levels and enhancing HRQOL and social support.^12^ Recently, MBIs were included in joint Society for Integrative Oncology-American Society of Clinical Oncology clinical practice guidelines for treating anxiety and depression symptoms in people with cancer, recommending that MBIs should be offered to people with cancer both during and after cancer treatments.^13^ Despite such encouraging evidence, accessing such programs can be challenging for patients. Frequent barriers can include limited mobility due to treatment-related side effects, geographical and time constraints, financial restraints, and cultural stigmas, particularly in low- and middle-income countries (LMICs).^14^

In light of these circumstances, researchers in Canada translated their evidence-based program into an app-based format.^15^ The Mindfulness-Based Cancer Survivorship Journey (MBCS) is hosted on the digital therapeutic platform AmDTx (Mobio Interactive PTE LTD), which includes innovative features like cognitive stress quantification through a 30-s selfie video captured with the smartphone camera.^16,17^ “AmDTx-MBCS” is a Class II Software as a Medical Device with Health Canada Investigational Testing Authorization and pending Food and Drug Administration (FDA) approval for commercialization in the United States.

Importantly, a recent systematic review highlighted the potential benefit of smartphone application-based MBIs. This innovative approach provides a cost-effective and accessible alternative for delivering psychosocial cancer care to patients.^18^ However, it is worth noting that although studies have demonstrated the efficacy of smartphone application-based MBIs in improving HRQOL and reducing emotional symptoms in patients with cancer, there remains limited research in advanced disease and less common malignancies.^19^ Few clinical trials have included patients with mRCC, for example, despite the known correlation between psychological well-being and survival time among patients with RCC, implying the potential for this psychosocial intervention to impact on not only emotional symptoms but also biological processes.^20^ Moreover, there is a scarcity of studies exploring the use of eHealth interventions in LMIC. Healthcare providers in these countries often encounter challenges in delivering such interventions due to limited training and technology resources.^14,21^ To ensure equity in providing psychosocial interventions to patients with cancer, it is crucial for researchers to investigate the feasibility and efficacy of these interventions while incorporating data from diverse countries and healthcare settings.

This multinational study sought to determine the feasibility and acceptability of a mindfulness app-based intervention among patients with mRCC who were receiving immunotherapy and experiencing clinical symptoms of anxiety. Additionally, the study aimed to explore the preliminary efficacy of this intervention on emotional and physical symptoms, trait mindfulness, and overall HRQOL. We hypothesize that the mindfulness app-based intervention will be feasible and acceptable among patients with mRCC receiving immunotherapy and will demonstrate preliminary efficacy in improving emotional and physical symptoms, trait mindfulness, and overall HRQOL.

## Methods

This is a longitudinal study performed at a National Cancer Institute-designated Comprehensive Cancer Care (Duarte) and at a linked network located across different Brazilian states (North, Northwest, Central-West, Southeast, and South) from June 2020 to January 2023. Eligible patients were diagnosed with mRCC, receiving immunotherapy, and experiencing clinically relevant symptoms of anxiety, defined as a PROMIS-Anxiety score of ≥ 60. In addition, patients were required to have a smartphone with internet access, not currently engage in meditation one or more times per week within the previous year, and have not participated in a mindfulness program in the past 5 years. The study protocol was reviewed and approved by the Ethics Review Board at the City of Hope and the Santa Marta Hospital, respectively.

This study was conducted in the United States and then culturally localized for evaluation in the Brazilian healthcare setting. Brazilian participants used the app in Portuguese, ensuring that the content was linguistically and culturally relevant. No additional modifications to the intervention content were made beyond. The AmDTx app, which contains the MBCS for use in clinical trials, is freely available for download from the Apple and Google app stores. The app operates on a Freemium model, which means that some features are free and others at a cost. For the purpose of this study, patients were given free access to all features included within AmDTx, including the MBCS intervention.

### Procedure and intervention

Eligible patients were invited to participate in the study through a combination of methods, including direct communication from their healthcare providers and distribution of study information pamphlets in clinical settings. If patients expressed interest, study staff followed up via phone to explain the study in detail. The consenting procedure was conducted either in person or via a secure online platform. Once consented, participants were provided instructions to download the AmDTx app from either the Apple or Google Play Store. Study staff offered support to assist participants with the download and setup process if needed. Outcome measures were completed over the phone.

Patients were encouraged to engage in mindfulness app-based activities for 20–30 min every day, for a minimum of 4 days per week, for 4 weeks. Each session was defined as one 20–30 minutes engagement with the app, which included activities such as guided meditation, psychoeducation on mindfulness principles, and journaling prompts. Participants were particularly encouraged to complete the 27-stage MBCS intervention, which consisted of individual lessons building on each other to enhance mindfulness skills.^15^ The intervention provides guided meditation exercises and suggestions for coping with cancer and cancer symptoms, modeled on the MBCR program and book.^11^ AmDTx-MBCS consists of 5 core modules, including an introduction to MBCS, mindful attitudes, stress responses, cognitive coping strategies, and mindful imagery. The meditation activities included body scans, mindfulness of breath, mindful movement, breathing exercises, walking meditation, open awareness, and compassion meditation.^15^ Weekly reminders, designed to promote adherence, were sent to participants as brief motivational messages (eg, “Don’t forget to take a few moments today for your mindfulness practice. Your well-being matters!”). The Principal Investigator (PI) was available for questions during the first 3 days after recruitment to ensure that participants understood how to use the app. No additional human support was provided beyond this initial availability.

Patients were assessed at 4-time points, baseline, and weeks 2, 4, and 12, for emotional symptoms (Patient-Reported Outcomes Measurement Information System-Anxiety, PROMIS-Depression, and Fear of Cancer Recurrence-7), fatigue (Brief Fatigue Inventory [BFI]), trait mindfulness (Mindfulness Attention Awareness Scale), and HRQOL (Functional Assessment of Chronic Illness Therapy-General). Before each assessment, patients were asked if they had encountered any issues with the app and whether they actively participated in the app-based activities.

### Outcome measures

#### *PROMIS* e*motional* d*istress*: anxiety

This 8-item measure assesses symptoms of anxiety on a 5-point scale (1 = never, 5 = always). Scores range from 7 to 35 with higher scores indicating greater severity of anxiety.^22,23^

#### *PROMIS emotional distress*: *depression*

This 8-item measure assesses symptoms of depression on a 5-point scale (1 = never, 5 = always). Scores range from 8 to 40 with higher scores indicating greater severity of depression.^22,23^

#### Fear of cancer recurrence-7

This is a 7-item scale that assesses the degree of FCR, with a cutoff score of 17 or above indicative of moderate and a cutoff score of 27 or above indicative of severe FCR.^6^ Total scores range from 6 to 45.^24,25^

#### Brief fatigue inventory

 This 9-item, 11-point rating scale was developed to assess subjective fatigue. The first 3 questions measure fatigue severity from 0, indicating “no fatigue,” to 10, indicating “as bad as you can imagine,” at current, usual, and worst levels. The following 6 questions assess fatigue interference with daily activities including general activity, mood, walking ability, normal work (both inside and outside the home), relationships with other people, and enjoyment of life. Response options range from 0, indicating “does not interfere,” to 10, indicating, “completely interferes.” Higher scores on the BFI correspond to greater self-reported levels of fatigue.^26^

#### Mindfulness attention awareness scale

A 15-item scale, designed to assess characteristics associated with mindfulness, such as open or receptive awareness of and attention to what is taking place in the present. Participants use a scale from 1 to 6 (almost always—rarely), to indicate how frequently or infrequently they have each experience. Higher scores reflect higher levels of dispositional mindfulness.^27,28^

#### Functional assessment of chronic illness therapy-general

A 27-item self-related scale measures HRQOL across 4 domains of “well-being” (physical, social/family, emotional, and functional) on a 4-point Likert scale. Scores range from 0 to 8 for the physical, social/family, and functional subscales, 0–24 for the emotional subscale, and 0–108 for the total score.^29,30^

### Statistical analysis

Descriptive statistics were calculated to summarize the baseline demographic and clinical characteristics of the patients and are presented as mean ± SD or frequency [*n*, %], as appropriate. An intention-to-treat analysis design was employed for this study, which means that all participants, including those who were non-adherent to the protocol, were included in the statistical analyses.

It is important to note that we did not observe significant differences in patient’s baseline characteristics between Brazil and the United States. As a result, we combined the data from both countries and presented the findings without conducting a direct comparison between the 2. This approach allowed us to analyze the collective results and provide a comprehensive overview of the intervention’s impact on the measured outcomes across the multinational patient population. By combining the data, we aimed to maximize the statistical power and generalizability of our findings, contributing to the limited existing research on mindfulness interventions in the context of mRCC treatment.

Feasibility was assessed by self-reported adherence, defined as ≥50% of patients completing a minimum of 3 out of 4 intended intervention sessions for at least 2 out of 4 weeks. A session refers to one 20–30-min activity (lesson completion) a day. Additionally, feasibility required that at least 70% of patients had at least 2 out of 4 evaluable time points for the 4 assessments conducted in this study. Acceptability was assessed through a survey with open-ended questions designed to provide insights into participant engagement and acceptance (“Please share your overall experience with the intervention. What aspects did you find appealing or engaging, and do you have any suggestions for improvement?”). These questions were administered at multiple assessment points throughout the study (baseline and at weeks 2, 4, and 12). Subsequently, the responses were transcribed and independently reviewed by 2 individuals (C.B., P.B.). The content was then categorized into specific domains related to their adherence level (high: 3 + days per week vs moderate to low: 2 or fewer days per week). In cases where discrepancies arose, discussions were held, and a consensus was reached through adjudication.

To evaluate changes in the outcomes (dependent variables) from baseline, a linear mixed model approach was utilized. The model was adjusted for fixed covariates, including age, gender, and race. Only complete cases with data available at T4 were included in this analysis. Additionally, to assess the effect size of the changes between T1 and T4, Cohen’s d was calculated. SPSS software was used for the statistical analysis, and a type I error probability of α=5% was set for determining statistical significance.

## Results

### Patient characteristics (Table 1)

Patients were recruited from 2 countries, Brazil (*N* = 30) and the United States (*N* = 20). The characteristics of the patients were well balanced between the 2 countries. Most patients were male (68.0%), with a median age of 59 years (range: 32–88). In terms of ethnicity, 64.0% of patients identified as White (64.0%). Most patients were married (74.0%) and had a college degree (64.0%). At the time of treatment, patients were primarily either retired (42.0%) or employed (38.0%). Most patients were diagnosed with clear cell histology (82.0%).

**Table 1.** Patients’ characteristics at baseline (*N* = 50).

Characteristics	*N* (%)/median (range)
*Sex* [*N*(%)]	
Male	34 (68.0)
Female	16 (32.0)
*Age* [*median* (*range*)]	59 (32–88)
*Race* [*N*(%)]	
White	32 (64.0)
Multiracial	9 (18.0)
Asian (including Chinese)	8 (16.0)
Black	1 (2.0)
*Marital status* [*N*(%)]	
Married	37 (74.0)
Single	7 (14.0)
Divorced	5 (10.0)
Widowed	1 (2.0)
*Education* [*N*(%)]	
High school	18 (36.0)
College degree	23 (46.0)
Beyond college (graduate or professional degree)	9 (18.0)
*Occupation* [*N*(%)]	
Retired	21 (42.0)
Employed	19 (38.0)
Disability	8 (16.0)
Other	2 (4.0)
*Histology* [*N*(%)]	
Clear cell	41 (82.0)
Papillary	7 (14.0)
Chromophobe	2 (4.0)

### Feasibility and acceptability (Table 2)

**Figure 1.**
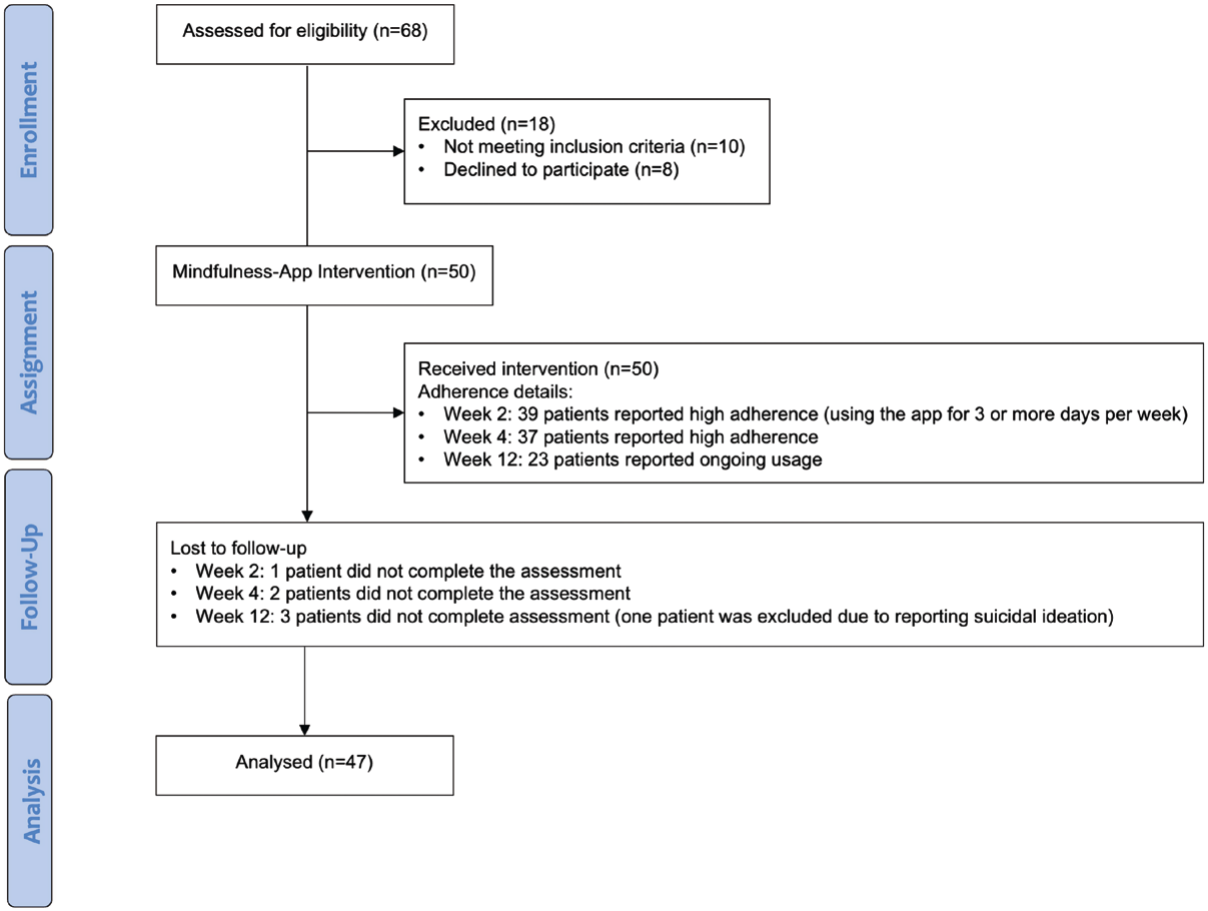
CONSORT flow diagram.

A total of 50 patients were recruited for this study (Figure 1). At week 2 (T2), 49 patients (98.0%) completed the assessments, while at week 4 (T3), 48 patients (96.0%) were assessed. By week 12 (T4), 47 patients (94.0%) had successfully completed the assessment.

**Table 2.** Patients’ quotes by adherence level.

Adherence level	Quotes	Adherence frequency
*High*		
T2	“I have been using it frequently with my wife. I really like it. It has been a great opportunity for me to reconnect with my body. After the diagnosis, I felt the desire to be free from my own body because it was sick.” “I have been using it frequently. It has helped me sleep better.”	77.5
T3	“I have been enjoying the app a lot. I have noticed an improvement in my anxiety and my concern about the next appointment.” “I feel that my fatigue has improved significantly.”	75.0
T4	“I have been practicing with my daughter. I really enjoyed the meditation during my walk. I have been feeling less anxious.” “I have been using it frequently. Sometimes I try to replicate the meditation practices when I listen to music.”	48.9
*Moderate to low*		
T2	“When I open the app, I receive an error message. I’ve been having difficulties in proceeding with the activities. I don’t know how to continue with the last module.” “I have been using it frequently, but the MBCS journey is not available for me.”	22.5
T3	“It’s interesting, but I haven’t noticed any difference yet.” “I use it whenever I remember.” “With the holiday season, I ended up forgetting.”	25.0
T4	“I lost my cellphone. I couldn’t download the app again.” “I haven’t been using it. My wife had a stroke. I am very worried about her.” “I haven’t been using it anymore.”	51.1

Overall, 26.0% of patients experienced difficulties in accessing the app during T2. However, this percentage decreased over time, with only 6.0% reporting issues at T3, and no reported issues at T4. The most common challenges encountered were related to difficulties in understanding how the app works (54.9%) and technical difficulties (46.1%). Notably, 24.0% of patients required alternative access methods to ensure effective app usage. These methods included providing new access codes or detailed troubleshooting instructions when technical issues or difficulties in understanding how to use the app. In cases where patients faced challenges understanding the app, their caregiver played a crucial role in providing guidance and support.

At week 2, 77.5% of patients reported a high level of adherence to the app, using it for 3 or more days per week. In contrast, 22.5% reported moderate to low adherence, using the app for 2 or fewer days per week. Patients with high adherence reported enjoyment in using the app and noted their perceptions of improvement in emotional and physical symptoms. One patient mentioned a reduction in their psychotropic medication for anxiety. Patients with moderate to low adherence cited reasons such as forgetting to use the app or not understanding the relevance of the intervention.

By week 4, 75.0% of patients reported a high level of adherence to the app and experienced improvement in symptoms. It is important to note that one patient was excluded at this time point due to reporting suicidal ideation, and appropriate psychiatric care was provided. Additionally, the patient who had previously reduced their psychotropic medication mentioned that they had completely stopped taking it under the guidance of their psychiatrist. Furthermore, 37.5% of patients reported that this intervention has helped them cope with waiting for test results and follow-up appointments. At week 12, 48.9% of patients self-reported ongoing usage of the app, indicating sustained engagement with the intervention. Overall, the study achieved a 77.5% rate of complete adherence at week 2 (use the app for 3 or more days for at least 2 out of 4 weeks).

### Outcome measures

Mixed model analyses revealed significant improvements over time in emotional symptoms, fatigue, trait mindfulness, and HRQOL (Table 3). The mean scores for anxiety (mean difference = 9.2, SE = 0.7, *P* = .001, Cohen’s d = 1.8), depression (mean difference = 4.7, SE = 0.5, *P* = .001, Cohen’s d = 0.9), fear of cancer recurrence/progression (mean difference = 7.8, SE = 1.0, *P* = .001, Cohen’s d = 1.5), and fatigue (mean difference = 13.6, SE = 2.7, *P* = .001, Cohen’s d = 0.7) significantly decreased from baseline (pre-intervention) to post-intervention (T4). Conversely, there was a significant increase in mean scores for trait mindfulness (mean difference = 12.5, SE = 1.5, *P* = .001, Cohen’s d = 1.0) and HRQOL (mean difference = 12.3, SE = 2.0, *P* = .001, Cohen’s d = 0.9).

**Table 3.** Change in outcomes over time: adjusted least square means in longitudinal mixed model analysis (*N* = 47).

Scale	Least square means (SD)				Difference (Time 4 vs Time 1)	SE	*P*-value	Cohen’s d
	Time 1	Time 2	Time 3	Time 4				
PROMIS-Anxiety	21.5 (5.1)	17.2 (5.8)	13.7 (5.5)	12.3 (5.0)	9.2	0.72	**.001**	1.82
PROMIS-Depression	14.9 (5.5)	11.7 (4.5)	10.8 (4.3)	10.1 (4.0)	4.7	0.59	**.001**	0.99
FCR-7	21.1 (4.5)	16.6 (5.2)	14.2 (5.5)	13.2 (5.6)	7.8	1.07	**.001**	1.55
BFI	31.0 (17.8)	27.8 (20.0)	21.0 (17.4)	17.4 (18.7)	13.6	2.73	**.001**	0.74
MAAS	56.2 (13.2)	61.2 (13.3)	66.0 (11.0)	68.7 (10.8)	12.5	1.58	**.001**	1.03
FACT-G	81.8 (13.1)	87.5 (12.9)	91.7 (13.4)	94.1 (13.8)	12.3	2.01	**.001**	0.91

Models were adjusted for the effects of age and gender.

Abbreviations: BFI, Brief Fatigue Inventory; FACT-G, Functional Assessment of Cancer Therapy-General; FCR-7, Fear of Cancer Recurrence-7; MAAS, Mindfulness Attention, Awareness Scale. Italic was used to indicate the variable, while bold was used to highlight significant findings.

## Discussion

This is the first study to investigate the feasibility of a mindfulness app-based intervention among patients with mRCC undergoing immunotherapy in both high-income and LMICs. Previous research on similar interventions has primarily focused on patients with localized cancer who have completed all cancer treatment, such as breast, prostate, or colorectal cancer, while few studies have explored the benefit of such interventions among patients with advanced or metastatic disease.^31,32^ Additionally, it is noteworthy that the majority of participants in our sample are male, which uniquely differs from the predominately female samples in most other studies.^33^

Our findings indicate that this smartphone-accessible psychosocial support tool is both feasible and acceptable among patients with mRCC in both Brazil and the United States. A systematic review encompassing 21 articles, consisting of studies conducted in the United States, Canada, Australia, Netherlands, United Kingdom, Denmark, Italy, Japan, and China, demonstrated the potential for mindfulness home practice interventions to improve emotional symptoms, including stress, anxiety, depression, and fear of cancer recurrence.^32^ However, adherence to home practice-based interventions was found to be suboptimal across multiple studies.^32^ Factors such as being married and higher social support have been associated with higher adherence rates, although research in this arena is ongoing. Our findings align with this trend, as patients who demonstrated adherence to the intervention frequently noted the support that they received from their family members. Additionally, the use of weekly reminders and the requirement of only 20 minutes per day for the intervention may have contributed to the patient’s acceptance and adherence. The convenience of the smartphone-based approach further facilitated greater acceptance and improved adherence rates among patients, particularly for those with metastatic cancer who often face additional barriers in accessing supportive care services. Further research is needed to understand the long-term impact of these app-based interventions, including the continuity of practice over time, although in the short term (12 weeks), we observed that over half of the patients continued practicing the intervention.

Notably, a significant decrease in emotional symptoms and fatigue scores was observed from baseline to post-intervention. The effect size, as indicated by Cohen’s d, was moderate for fatigue and large for emotional symptoms. This suggests that this intervention had a positive effect on alleviating these symptoms. The reduction in emotional symptoms is particularly important, as patients with Renal cell carcinoma (RCC) often experience heightened levels of distress and fear of cancer recurrence during their treatment journey.^1,34,35^ It is important to highlight that emotional symptoms are often underdiagnosed and undertreated in this patient population, with limited studies exploring psychosocial support interventions to manage such symptoms specifically tailored for patients with RCC. For instance, a previous study demonstrated the benefit of expressive writing for patients with RCC in mitigating cancer-related symptoms and improving HRQOL.^36^ However, beyond this study, research on psychosocial support intervention specifically tailored for patients with RCC remains scarce. Notably, patients with mRCC can face a multitude of challenges, including the uncertainty and fear associated with this diagnosis and prognosis, as well as the increasing array of available treatments and the associated burden of decision-making. Treatment-related side effects remain another significant challenge, with fatigue being highly prevalent and both physically and emotionally taxing.^37^

Furthermore, a significant increase was noted for trait mindfulness and HRQOL scores following the intervention, with effect sizes being large for both outcomes. This suggests the intervention enhanced their ability to be fully present and engaged. Mindfulness is an effective strategy for managing distress, promoting emotional well-being, and enhancing overall HRQOL in patients with cancer.^38^ These findings support the potential benefits of incorporating this low-cost intervention into the standard care of patients with cancer. By addressing emotional symptoms, fatigue, and promoting mindfulness, healthcare providers can improve the holistic care of their patients and enhance their overall treatment outcomes. It is important to note that the intervention was well-accepted by patients, as indicated by the high adherence rates observed.

However, there are some limitations to consider. First, the sample size of our study was relatively small, which may limit the generalizability of our findings to a larger population. Future research with larger sample sizes is needed to confirm and expand upon our results. Second, our study focused on a specific patient population, and thus the results may not be applicable to all cancer types, disease stages, and treatment modalities. Moreover, the study involved participants from 2 distinct healthcare systems: a comprehensive cancer center in the United States and a diverse healthcare network in Brazil. Variations in access to healthcare, insurance, and regional practices could impact the app’s implementation and effectiveness, potentially influencing the study outcomes. Furthermore, it is worth noting that our sample consisted primarily of highly educated patients, which could introduce some level of bias. Moreover, adherence levels were determined based on patients’ self-reported data, which carries the possibility of introducing bias. Fourth, the short-term follow-up in our study may not fully capture the long-term effects of the MBI. Future studies with extended follow-up periods are essential to understand the sustained impact of MBIs among patients with mRCC. Fifth, the reliance on self-reported data for assessing app engagement. Consequently, self-reported adherence and engagement may not fully reflect participants’ true interaction with the app. Future studies should address these limitations by implementing more robust tracking systems and exploring alternative methods for verifying engagement to enhance the reliability of the data. Additionally, by including only complete cases with data available at T4, there is a risk of selection and guarantee-time biases, which may affect the representativeness of the results. Future research should consider employing intent-to-treat analyses to better address these biases. Finally, the absence of a control group in our study limits our ability to establish causality between the mindfulness intervention and the observed outcomes. Although our findings suggest positive effects, randomized clinical trials with control groups will be necessary to assess the interventions’ efficacy. In conclusion, further research is warranted to explore the effectiveness of MBIs among patients with localized RCC and those undergoing different types of treatment.

In conclusion, our study highlights the potential benefits of incorporating a low-cost mindfulness app-based intervention into the standard care of patients with mRCC in both high-income and LMICs. The intervention showed promise in reducing emotional symptoms, fatigue, and improving HRQOL and trait mindfulness in patients with cancer. Future research should further investigate the long-term effects of the intervention and its applicability in patients undergoing different types of treatment and patients with localized RCC.

## Data Availability

The data that support the findings of this article are available from the corresponding author upon reasonable request.
